# Metabolite pathway alterations identified by magnetic resonance metabolomics in a proximal tubular epithelial cell line treated with TGF‐β1

**DOI:** 10.14814/phy2.70249

**Published:** 2025-02-16

**Authors:** Tyrone L. R. Humphries, Soobin Lee, Aaron J. Urquhart, David A. Vesey, Aaron S. Micallef, Clay Winterford, Andrew J. Kassianos, Graham J. Galloway, Ross S. Francis, Glenda C. Gobe

**Affiliations:** ^1^ Kidney Disease Research Collaborative The University of Queensland and Translational Research Institute Brisbane Queensland Australia; ^2^ School of Biomedical Sciences, Macgregor Building The University of Queensland St Lucia Queensland Australia; ^3^ Department of Kidney and Transplant Services Princess Alexandra Hospital Woolloongabba Queensland Australia; ^4^ Central Analytical Research Facility Queensland University of Technology Brisbane Queensland Australia; ^5^ QIMR‐Berghofer Medical Research Institute Brisbane Queensland Australia; ^6^ Conjoint Internal Medicine Laboratory, Chemical Pathology Pathology Queensland Brisbane Queensland Australia; ^7^ Herston Imaging Research Facility The University of Queensland Herston Queensland Australia

**Keywords:** 2D‐COSY, metabolomics, metabonomics, NMR, nuclear magnetic resonance, proximaltubular epithelial cell, TGF‐β1, two‐dimensional correlated spectroscopy

## Abstract

Tubulointerstitial fibrosis is a characteristic hallmark of chronic kidney disease (CKD). Metabolic perturbations in cellular energy metabolism contribute to the pathogenesis of CKD, but the chemical contributors remain unclear. The aim of this investigation was to use two dimensional ^1^H‐nuclear magnetic resonance (2D‐COSY) metabolomics to identify the chemical changes of kidney fibrogenesis. An in vitro transforming growth factor‐β1 (TGF‐β1)‐induced model of kidney fibrogenesis with human kidney‐2 (HK‐2) proximal tubular epithelial cells (PTEC) was used. The model was validated by assaying for various pro‐fibrotic molecules, using quantitative PCR and Western blotting. 2D‐COSY was performed on treated cells. Morphological and functional changes characteristic of tubulointerstitial fibrosis were confirmed in the model; expression of fibronectin, collagen type IV, smooth muscle actin, oxidative stress enzymes increased (*p* < 0.05). NMR metabolomics provided evidence of altered metabolite signatures associated with glycolysis and glutamine metabolism, with decreased myo‐inositol and choline, and metabolites of the oxidative phase of the pentose phosphate pathway with increased glucose and glucuronic acid. The altered PTEC cellular metabolism likely supports the rapid fibrogenic energy demands. These results, using 2D‐COSY metabolomics, support development of a biomarker panel of fibrosis detectable using clinical magnetic resonance spectroscopy to diagnose and manage CKD.

## 1 INTRODUCTION

Chronic kidney disease (CKD) is a major cause of morbidity and mortality in global populations, and its prevalence is rising. CKD is defined by evidence of kidney damage (structural or proteinuria) and/or reduced kidney function, usually as proteinuria or estimated glomerular filtration rate (eGFR), for more than 3 months (Chen et al., 2019; Jha et al., 2013). The CKD prevalence rate of over 10% of the adult population exceeds acceptable disease prevalence levels in a general population (Jha et al., 2013). As the burden of disease grows, there is increasing urgency to develop sensitive biomarkers for early diagnosis of CKD and to monitor its progression.

The kidney is susceptible to various injuries that originate in glomerular, vascular, and/or tubulointerstitial compartments that create an environment conducive for CKD development. Tubulointerstitial fibrosis is a basic pathological manifestation of CKD (Agarwal & Nath, 2020; Hewitson et al., 2017). Maladaptive tissue repair following prolonged kidney injury can result in dysregulated fibrosis, which is characterized by tubular atrophy, chronic inflammatory cell infiltration, and tissue scarring characterized by the accumulation of extracellular matrix (ECM) proteins, such as fibronectin and collagen type I and IV (Agarwal & Nath, 2020; Gewin et al., 2017). A foundational study involving the histological analysis of kidneys from ischemic mouse models and failed human kidney transplants indicated that fibrosis, or more specifically tubulointerstitial fibrosis, correlated closely with the functional decline of kidneys and was a consistent pathological manifestation of CKD, irrespective of disease etiology (Fox, 1967).

The development of tubulointerstitial fibrosis is a complex multifactorial process mediated by crosstalk between different cell populations within the kidney itself, including endothelial and, fibroblasts, and tubular epithelial cells (TEC), and both tissue‐resident and circulating immune cells. The latter have been recognized as key cellular mediators of fibrosis and inflammation (Gewin, 2018; Qi & Yang, 2018). Persistent kidney injury triggers a cascade of cellular responses resulting in the release of pro‐inflammatory growth factors, such as, tumor necrosis factor (TNF) and pro‐fibrotic cytokines, such as transforming growth factor‐β1 (TGF‐β1) and connective tissue growth factor (CTGF). TGF‐β1 is a key contributor to progression of kidney fibrosis (Hills et al., 2018; Kim et al., 2018; Meng et al., 2016) and its inhibition reduces development of fibrosis in the kidney after injury (Moon et al., 2006). Under the influence of such molecules, PTEC are modified to mediate and exacerbate fibrosis. One characteristic fibrogenic change of PTEC is epithelial‐mesenchymal transition (EMT), whereby the PTEC undergo cytoskeletal rearrangement to phenotypically reflect myofibroblast‐like cells (Grande et al., 2015; Lovisa et al., 2015; Lu et al., 2011; Mack & Yanagita, 2015). This may be characterized by decreased expression of the epithelial cell marker E‐cadherin and increased expression of the mesenchymal markers of α‐smooth muscle actin (α‐SMA) and vimentin. Myofibroblasts, in turn, secrete excessive levels of ECM proteins, producing a network of fibrous structural proteins such as collagen type I and IV and elastin, and adhesive proteins such as fibronectin and proteoglycans (Grande et al., 2015; Lovisa et al., 2015). There is some contention surrounding the extent to which EMT influences fibrogenesis in the kidneys. For example, Lovisa et al. found that while some PTEC undergo phenotypical changes, they do not leave the basement membrane and a complete conversion to a mesenchymal phenotype is rare. Nevertheless, damaged PTEC exhibiting partial or whole EMT still perpetuate chronic fibrosis through cell cycle arrest and decreased functional parenchyma (Lovisa et al., 2015).

The effect of TGF‐β1 on other epithelial cell types in the nephrons has not been explored as extensively as in PTECs; however, it is likely that there are additional effects specific to their function and composition. For example, TGF‐β1 has an inhibitory effect on epithelial sodium channels that are mostly located in the distal convoluted tubules and the collecting ducts of the nephron (Xu et al., 2022).

The increase in changes associated with fibrosis suggests a need for greater energy demand to support the fibrotic process. A recent in vitro study demonstrated that human primary PTEC, originating from fibrotic human kidney, functioned differently from those of healthy kidneys in response to oxidative stress, a known contributor to fibrosis (Khan et al., 2020). Normal healthy kidney cells had significantly reduced mitochondrial function and increased cell death (necrosis) under oxidative stress, whereas PTEC from fibrotic kidneys were resistant to oxidative stress, with maintenance of mitochondrial function and cell viability. Thus, PTEC under a profibrotic influence may demonstrate altered energy metabolism that allows for their production of ECM proteins and their differentiation and maintenance as chronic pro‐inflammatory cells, like myofibroblasts, in an adverse profibrotic environment.

Metabolic reprogramming has been recognized as a critical characteristic of cancer cells to support greater energy requirements because of increased cellular proliferation and growth (Hua et al., 2020; Yu et al., 2020). Although it is likely that energy demand of a healthy kidney is altered and reprogrammed in a pro‐fibrotic environment (Mount & Power, 2015), currently there is limited understanding about altered metabolism in kidney fibrosis. Metabolomics, through various methods, can be used to elucidate these metabolic perturbations (Abbiss et al., 2019; Hwang et al., 2010; Khattri et al., 2021). The application of metabolomics has been used in the discovery of altered chemicals in a range of kidney diseases such as acute kidney injury (AKI), CKD, and loss of kidney function as a complication of diabetes mellitus and diabetic nephropathy (Abe et al., 2013; Afshinnia et al., 2018; Jang et al., 2020; Kang et al., 2015; Lan et al., 2016; Lee et al., 2018; Qiu et al., 2015; Srivastava et al., 2018). With metabolites typically measured from by‐products of whole‐body systems such as serum, plasma, or urine, fibrosis induced in PTEC in the whole kidney and the exclusive influence of specific metabolites on metabolic dysregulation in fibrosis, are difficult to assess. Metabolite profiles are measured with an analytical technique commonly used in metabolomics termed proton nuclear magnetic resonance (^1^H‐NMR) spectroscopy (Khattri et al., 2021; Kim et al., 2014; Zhang et al., 2012). The non‐invasive nature of ^1^H‐NMR spectroscopy enables its consideration for in vivo clinical applications in the form of magnetic resonance spectroscopy; hence, the selection of this technique serves a long‐term translational goal (Psihogios et al., 2007; Ramadan et al., 2012).

The current investigation sought to investigate metabolic changes that are occurring in proximal tubular epithelial cells while under the influence of a powerful profibrotic cytokine. This model emulates a crucial step in the fibrogenic process which largely contributes to the progression of CKD. Moreover, we also highlight the utility of in‐cell NMR metabolomics for identifying these metabolic changes.

## 2 MATERIALS AND METHODS

### 2.1 Materials

All chemicals and tissue culture requirements were of high quality and purity, and unless otherwise indicated, were obtained from Sigma‐Aldrich (St. Louis, MO, USA), Thermo Fisher Scientific (Waltham, MA, USA), or Gibco/Life Technologies (Paisley, UK).

### 2.2 Cell culture of HK‐2 cells with TGF‐β1 treatment

The human kidney‐2 (HK‐2) cell line (American Type Culture Collection, Rockville, MA, USA) consists of immortalized adult normal kidney PTEC, based on histochemical, cytochemical, immune, and functional characteristics (Ryan et al., 1994). HK‐2 cells were maintained in Dulbecco's modified Eagle's medium/Ham's F12 (DMEM/F12, Gibco, catalogue #11320033) with 10% fetal bovine serum (FBS, Gibco, catalogue #10099141) and 1% penicillin–streptomycin (Gibco, catalogue #15140122). Cells were grown in a humidified atmosphere of 95% air and 5% carbon dioxide at 37°C in a tissue culture incubator. Following seeding for experiments at 1 × 10^4^ cells/mL and growth to 60% cell confluency, the cells were synchronized in serum‐free DMEM/F12 for 24 h. The cells were then subjected to treatment with vehicle control (serum‐free DMEM/F12; 0 ng/mL TGF‐β1) or 1, 5, or 10 ng/mL recombinant human TGF‐β1 (R&D Systems; Minneapolis, Minnesota, USA, catalogue #RDS240B010CF) in serum‐free medium for 24, 48, and 72 h. The outcomes of TGF‐β1 dose–response, compared with vehicle controls, were examined to determine an optimal treatment timepoint and concentration for the NMR experiments. The greatest fold‐change differences in mRNA transcript or protein expression levels of fibrogenesis molecules and changes to cellular morphology that indicated epithelial‐mesenchymal phenotypic transition were considered.

### 2.3 qPCR for gene expression in HK‐2 cells after TGF‐β1 treatment

The change in expression of fibrogenesis‐associated genes were investigated using quantitative polymerase chain reaction (qPCR) (Humphries et al., 2021; Vesey et al., 2013). The genes of interest were ECM proteins, fibronectin (FN) and collagen type IV alpha 1 (COL4A1), mesenchymal cell‐associated protein, alpha smooth muscle actin (α‐SMA/*ACTA2*), and enzymes involved in oxidative stress/fatty acid oxidation (FAO), peroxisomal acyl‐coenzyme A oxidase 1 (ACOX1) and acetyl‐coA acyltransferase 1 (ACAA1). 18 s ribosomal RNA (*18S rRNA*) was also measured as a reference housekeeping gene. At the end of each treatment timepoint, RNA was collected using the PureLink RNA Mini Kit (Invitrogen/Thermo Fisher Scientific, catalogue #12183018A) and supplier methods. Concentration yield was calculated using the NanoDrop Lite Spectrophotometer (Thermo Fisher Scientific). Subsequently, cDNA was synthesized from the RNA samples by reverse transcription, using the High‐Capacity cDNA Reverse Transcription Kit (Applied Biosystems/Thermo Fisher Scientific; Waltham, MA, USA, catalogue #4374967). The samples underwent primer annealing at 25°C for 10 min, DNA polymerization at 37°C for 120 min, and enzyme deactivation at 85°C for 5 min as per kit protocol. The cDNA was stored at −20°C until qPCR was performed using the QuantiNova SYBR Green PCR kit (QIAGEN/Life Technologies; Carlsbad, CA, USA, catalogue #208052) and a Lightcycler 480 system (Roche, Basel, Switzerland). All primers (Table 1) were supplied by IDT Australia (Boronia, Australia) except for the 18 s rRNA primer, which was supplied by Invitrogen/Thermo Fisher. The ΔΔCt method was employed to measure relative fold gene expression of the target genes from the reference gene 18 s rRNA. The final measures of gene expression for varying concentrations of TGF‐β1 treatment were calculated as a fold change from the vehicle control (0 ng/mL TGF‐β1) at each timepoint. A “no template control” (RNase‐free water) was included to serve as a control for RNA contamination and primer dimer formation.

**TABLE 1:** Forward and reverse primer sequences for target genes.

Primer	Primer sequence
FN	F: 5′‐CCC TGG TGT CAC AGA GGC TA – 3′ R: 3′‐TGT ATA TTC GGT TCC CGG TTC – 5′
COL4A1	F: 5′‐CAA GGG CTC GCC GGG TTC TG – 3′ R: 3′‐CCG GTG TCA CCA CGA CTG CC – 5′
ACTA2	F: 5′‐ATC ACC AAC TGG GAC GAC AT – 3′ R: 3′‐GGC AAC ACG AAG CTC ATT G – 5′
ACOX1	F: 5′‐CCA GGT AGT AAA AGC CTT CAG C – 3′ R: 3′‐GTA TAA ACT CTT CCC GCT CCT – 5′
ACAA1	F: 5′‐CAG GTT GTC ACG CTA CTC AA – 3′ R: 3′‐G AAA CTT ATG GGA CCC TTG ACT – 5′
18 s rRNA	F: 5′‐GTA ACC CGT TGA ACC CCA TT – 3′ R: 3′‐CCA TCC AAT CGG TAG TAG CG – 5′

Abbreviations: ACAA1, acetyl‐coA acyltransferase 1; ACOX1, peroxisomal acyl‐coenzyme A oxidase 1; ACTA2, smooth muscle actin; COL4A1, collagen Type 4A1; FN, fibronectin.

### 2.4 Western blots for secreted ECM protein changes from HK‐2 cells after TGF‐β1 treatment

Western blots of cell supernatants were used to quantify the expression of the ECM protein, fibronectin, and a tissue remodeling enzyme, plasminogen activator inhibitor‐1 (PAI‐1). HK‐2 cells were seeded and treated as previously described above for qPCR. The cell medium was harvested at each time point and TGF‐β1 concentration. Equal volumes of cell medium were diluted in a reducing Bolt LDS sample buffer (Invitrogen, catalogue #B0007) and heated to 70°C for 10 min. The protein ladder consisted of 5 μL SeeBlue Plus2 protein standard (Thermo Fisher Scientific, catalogue #LC5925) and 1 μL MagicMark XP protein standard (Thermo Fisher Scientific, catalogue # LC5602). Proteins were separated on a 4–12% NuPAGE gel (Thermo Fisher Scientific, catalogue #NP0322BOX) and transferred to 0.4 μm polyvinylidene difluoride membrane (Thermo Fisher Scientific, catalogue #88520). Membranes were blocked with PBST for 1 h before incubation with primary antibodies. Primary antibodies were supplied by Santa Cruz Biotechnology Inc. (Heidelberg, Germany), and secondary antibodies were supplied by R&D Systems Australia Pty Ltd. (Albion, Australia). Western blots were imaged using a ChemiDoc MP imaging system (Bio‐Rad, CA, USA). The densitometric analysis of protein band intensities was performed using Image J v. 2.3 software. Regions of interest (ROI) were placed around individual bands, and pixel intensity was measured using the “Analyze > Measure” function. ROIs were kept consistent for each protein. Band intensities were background corrected and normalized to the control.

### 2.5 Assessment of metabolite changes in HK‐2 cells treated with TGF‐β1 using ^1^H‐NMR

From the results of these assays, 10 ng/mL of TGF‐β1 for 48 h was selected for the ^1^H‐NMR analysis of kidney fibrogenesis.

HK‐2 cells were seeded and grown to 60% confluency in four T‐175 flasks. Note that two T‐175 flasks at sub‐confluency were needed to provide sufficient cell numbers for a pellet large enough to analyse using ^1^H‐NMR. The cells were serum‐starved for 24 h. Two flasks were treated with serum‐free DMEM/F12 (vehicle control), and two were treated with 10 ng/mL TGF‐β1 for 48 h. The cells were then dissociated with Accutase Cell Detachment Solution (Sigma‐Aldrich, catalogue #A6964), centrifuged, and the pellets washed twice with PBS to completely remove any exogenous metabolites in the cell medium. Supernatants were discarded after each wash. The pellet was resuspended in 10 mL of deuterium oxide/PBS (D_2_O/PBS; Sigma‐Aldrich, catalogue #1.13366) stabilizing solution. The cell suspension was centrifuged, and the pellet resuspended in 100 μL D_2_O/PBS, to make a high‐density cell suspension. Equivalent cell numbers were used in each NMR assay.

The D2O/PBS suspensions were pipetted into low‐volume Shigemi NMR tubes (Shigemi, Japan, catalogue #SP‐405) with magnetic susceptibility matched to D2O. Each tube was inserted into the Avance III HD 400 MHz NMR spectrometer equipped with a BBO probe (5 mm) with z‐gradients (1H: 400.16 MHz) (Bruker Biospin GmbH, Karlsrhule, Germany; Bruker Topspin 3.6.1. software) (Queensland University of Technology, Gardens Point, Australia). Assays were run in triplicate, giving *n* = 3 sets of data for each of control or treated cells. For each sample, the spectrometer was locked to the deuterium signal of the solvent, the probe tuned and matched to the 1H frequency and the magnet shimmed to ensure optimal magnetic field homogeneity. 2‐Dimensional COrrelated SpectroscopY (2D‐COSY) NMR spectra were acquired with water suppression (presaturation) for each sample at 37°C (Del Vecchio et al., 2022; Urquhart et al., 2023). Data were acquired by collection of 2k data points over a spectral width of 4000 Hz (10.0 ppm) with an acquisition time of 256 ms and 256 increments in the second dimension. For each increment, 4 averages were collected, to increase signal‐to‐noise, giving a total acquisition time of 39 min and 42 s.

With a post‐acquisitional processing pipeline developed by us (Del Vecchio et al., 2022; Urquhart et al., 2023) using Mnova v. 14.1.2 software (Mestrelab Research, Santiago, Spain), comparisons were made between vehicle control and treatment groups. Sequentially, apodisation across the t2 and t1 domain was used to optimize the triglyceride signals and reduce noise levels across the spectra, as previously described (Del Vecchio et al., 2022; Delikatny et al., 1991). To standardize the spectral analysis, each 2D‐COSY spectrum was calibrated on the chemical shift of choline at 3.23, 3.23 ppm (parts per million) signal. Following spectral processing and calibration, all signals of interest were identified and selected manually to create a template for integration to ensure a maximal signal of interest, while minimizing noise signal. The same template was applied to all spectra, and the integrated peak volumes were calculated. Using a cut‐off signal‐to‐noise ratio of >5, 82 cross‐peaks were identified, with the peak chemical shift ppm and integrated peak volumes collected for further analysis. The integrated peak volume values were processed with total intensity sum normalization, whereby intensity value for each spectroscopic feature is divided by the total sum within sample. Following total sum normalization of peak integrals, the dataset was further analyzed to identify the metabolite profiles which differed between the untreated and treated HK‐2 samples. Peaks corresponding to different chemicals were identified by collating with data available from the Human Metabolome Database (Del Vecchio et al., 2022; Wishart et al., 2022) and other referenced publications. Occasionally, a chemical could not be identified, and it was recorded as “undefined”.

### 2.6 Statistical analysis

The univariate statistical analyses for establishing the fibrogenesis model were conducted using Prism 9 (GraphPad Inc., USA). Data were logarithmically transformed to ensure parametric distributions. Normality was confirmed with the Shapiro–Wilk test, with a significance threshold of *p* ≤ 0.05. Significance was also evaluated using either an unpaired Student's *t*‐test with Welch's correction for two groups or a two‐way ANOVA, with a significance threshold of *p* ≤ 0.05, followed by Tukey's multiple comparisons test for three or more groups. All data were presented as mean ± standard deviation (SD). All figures were created using Prism 9 or BioRender (BioRender Inc., Canada). SIMCA‐16 software was used to perform multivariate statistics for Principal Component Analysis (PCA) and Orthogonal Projections to Latent Structures Discriminant Analysis (OPLS‐DA). OPLS‐DA is a supervised analysis which uses the trends identified in the PCA, and models data into pre‐assigned groups. Both modeling methods were used to provide insights into which chemicals contributed to the separations between untreated and treated groups. The resonances with Variable Importance for the Projection (VIP) scores, identified by multivariate statistics and VIP ≥1.2 (Del Vecchio et al., 2022; Ramadan et al., 2012; Urquhart et al., 2023), were selected for further univariate statistics using t‐tests for significance between control and experimental conditions.

## 3 RESULTS

### 3.1 TGF‐β1 increased mRNA transcript expressions of fibrogenesis‐associated molecules

**FIGURE 1:**
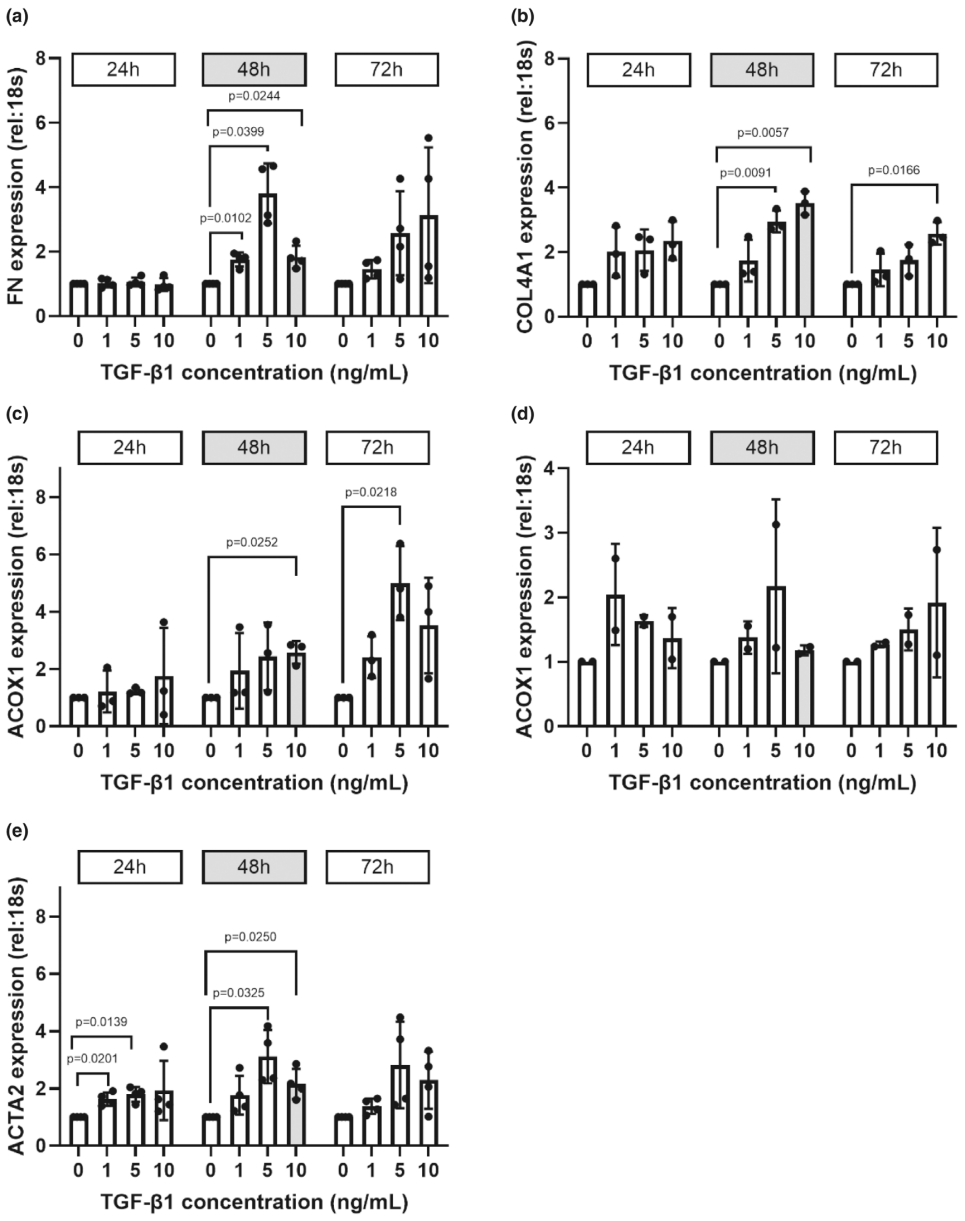
TGF‐β1 increased expression of fibrogenesis‐associated genes. Using qPCR, relative expression of target genes to the mean of the reference gene (18 s rRNA) was measured. Target genes investigated were (a, b) extracellular matrix proteins fibronectin (*FN*) and collagen type IV alpha 1 (*COL4A1*); (c, d) enzymes involved in fatty acid oxidation pathway, peroxisomal acyl‐coenzyme A oxidase 1 (*ACOX1*) and acetyl‐CoA acyltransferase 1 (*ACAA1*); and (e) mesenchymal cell‐associated protein alpha smooth muscle actin (*ACTA2*). They were compared at 24, 48, and 72 h at 1, 5, and 10 ng/mL TGF‐β1. Shaded columns and timepoint indicate the TGF‐β1 concentration and treatment time selected for downstream NMR analysis. Gene expression is shown as a mean fold change ± SD from vehicle control (0 ng/mL). Individual significant comparisons (compared to the 0 ng/mL) are annotated. *n* = 4 for FN and ACTA2, *n* = 3 for ACOX1 and COL4A1, *n* = 2 for ACAA1.

qPCR was performed to study the mRNA transcript expression of fibrogenesis‐indicative molecules of ECM proteins, FN and COL4A1, enzymes involved in the FAO pathway, ACOX1 and ACAA1, and mesenchymal cell‐associated protein ACTA2 at 24, 48, and 72 h TGF‐β1 treatment, at increasing concentrations of 1, 5, and 10 ng/mL TGF‐β1. Figure 1 indicates higher mRNA transcript expression of all five genes of interest with increasing TGF‐β1 concentrations and longer treatment periods. The mRNA levels were measured in relation to the reference gene 18 s rRNA, and within each timepoint, all samples were normalized to the vehicle control (0 ng/mL TGF‐β1). mRNA levels of FN and COL4A1 (Figure 1a,b) were significantly increased after TGF‐β1 treatment for FN, 2‐4‐fold increased expression at 48 h with 1 ng/mL (*p* = 0.0102, two‐way ANOVA, *n* = 4), 5 ng/mL (*p* = 0.0399), and 10 ng/mL (*p* = 0.0244); for COL4A1, 2‐4‐fold increased expression at 48 h with 5 ng/mL (*p* = 0.0091, two‐way ANOVA, *n* = 3) and 10 ng/mL (*p* = 0.0057) and 72 h with 10 ng/mL (*p* = 0.0166). The mRNA of enzymes involved in the FAO pathway, ACOX1 and ACAA1 (Figure 1c,d), showed similar increasing trends with TGF‐β1 treatment. For ACOX1, there was a 3‐5‐fold increase at 48 h with 10 ng/mL (*p* = 0.0252, two‐way ANOVA, *n* = 3) and at 72 h with 5 ng/mL (*p* = 0.0218). For ACAA1, there was a 1‐2‐fold increase (*n* = 2) from vehicle control across all treatment timepoints and TGF‐β1 concentrations; however, this analysis was underpowered and did not reach statistical significance. The transcripts for ACTA2 (Figure 1e) had a 1‐3‐fold increased level at 24 h with 1 ng/mL (*p* = 0.0201, two‐way ANOVA, *n* = 4) and 5 ng/mL (*p* = 0.0139) and 48 h with 5 ng/mL (*p* = 0.0325) and 10 ng/mL (*p* = 0.0250).

### 3.2 TGF‐β1 increased secretion of fibrogenesis‐associated proteins from treated HK‐2 cells

**FIGURE 2:**
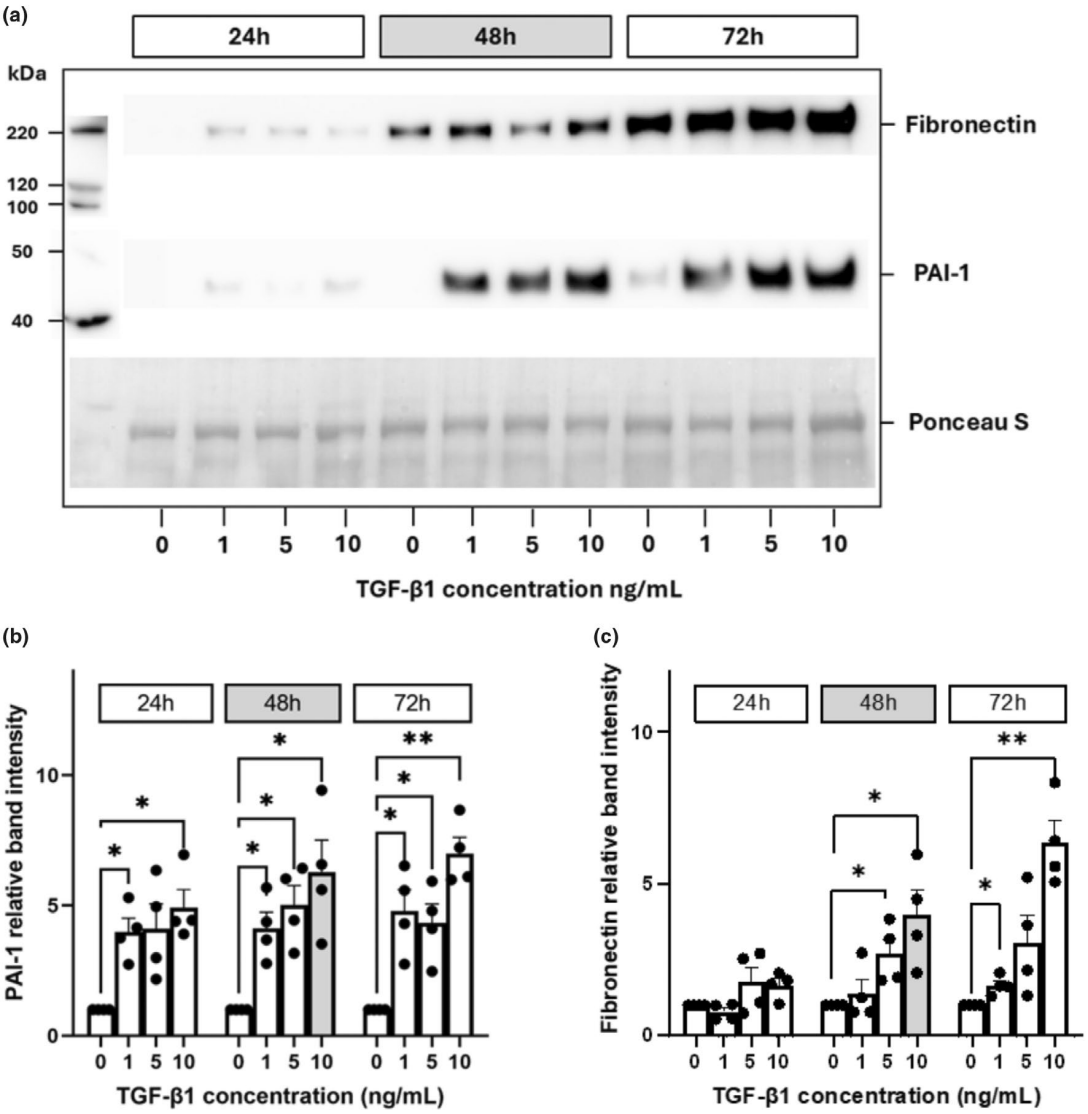
TGF‐β1 increased fibronectin and PAI‐1 protein in culture supernatant. Cell supernatant was obtained from cultured HK‐2 cells at different timepoints following treatment (24, 48, and 72 h) with TGF‐β1 (vehicle control 0; 1, 5, and 10 ng/mL). Representative Western blots are demonstrated in (a), the protein ladder is represented along the left‐hand side. The protein band intensity for PAI‐1 (b) and fibronectin (c) was normalized to total protein loading (Ponceau S) for each sample, and within each timepoint, all samples were normalized to the vehicle control. Data were logarithmically transformed and analysed using two‐way ANOVA with Tukey's multiple comparisons test. Bars show mean fold change in band intensity ± SD (*n* = 4). *p < 0.05; **p < 0.01.

Western blots were used to quantify the secreted proteins in cell media of the ECM protein, fibronectin, and tissue remodeling enzyme, PAI‐1, at 24, 48, and 72 h treatment, at increasing concentration of 1, 5, and 10 ng/mL TGF‐β1. Total protein loading for each sample was determined using Ponceau S stain. Figure 2 demonstrates the results. Representative blots in Figure 2a indicate higher fibronectin and PAI‐1 expression with longer treatment times and increasing TGF‐β1 concentrations. For densitometry (Figure 2b,c), treatments were normalized to the vehicle control. TGF‐β1 induced a significant increase in fibronectin (Figure 2b) and PAI‐1 (Figure 2c), with increased expression of fibronectin at 48 h and 5 ng/mL (*p* = 0.0446, two‐way ANOVA, *n* = 3) and 10 ng/mL (*p* = 0.0315), and at 72 h with 1 ng/mL (*p* = 0.0447) and 10 ng/mL (*p* = 0.0015). PAI‐1 was increased with 1, 5, and 10 ng/mL TGF‐β1 at 24 h (*p* = 0.0066, 0.0353, 0.0035, respectively), 48 h (*p* = 0.0083, 0.0070, 0.0097, respectively), and 72 h (*p* = 0.0122, 0.0142, 0.0007, respectively). Thus, at 48 h and a treatment concentration of 10 ng/mL there were statistically significant increases in both proteins.

### 3.3 ^1^H‐NMR analysis: TGF‐β induced changes in chemical profiles identified from 2D‐COSY spectra

**FIGURE 3:**
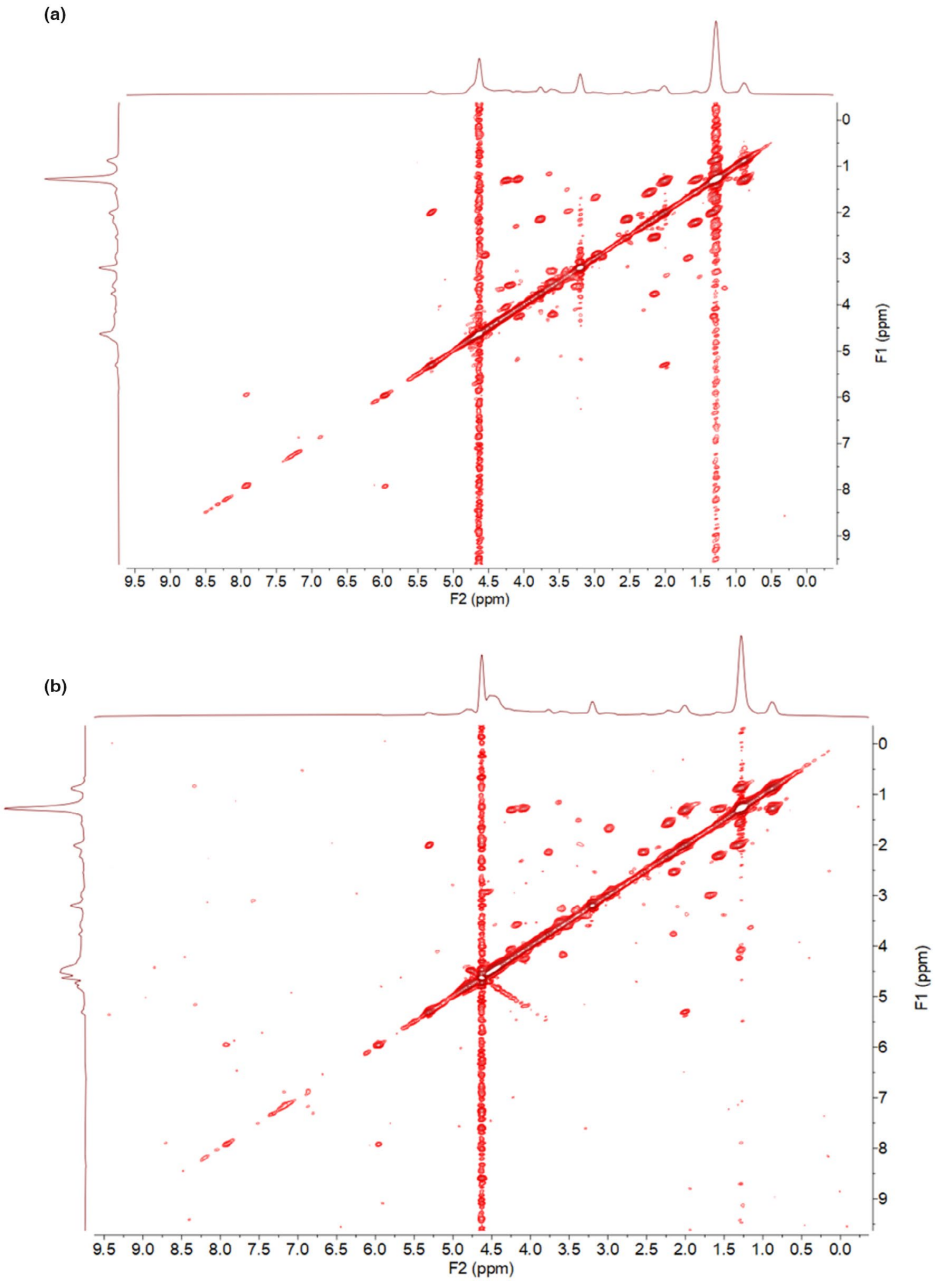
Representative 2D COSY spectra from vehicle control and TGF‐β1‐treated HK‐2 cells. Examples show two‐dimensional correlated spectroscopy (2D‐COSY) for (a) vehicle control (0 ng/mL) and (b) TGF‐β1 treatment (10 ng/mL) at 48 h treatment.

2D‐COSY spectra were used to profile alterations in metabolites. Figure 3a,b demonstrate representative spectra from 2D‐COSY ^1^H‐NMR for the vehicle control (A; 0 ng/mL) and treatment (B; TGF‐β1 10 ng/mL) at 48 h.

**FIGURE 4:**
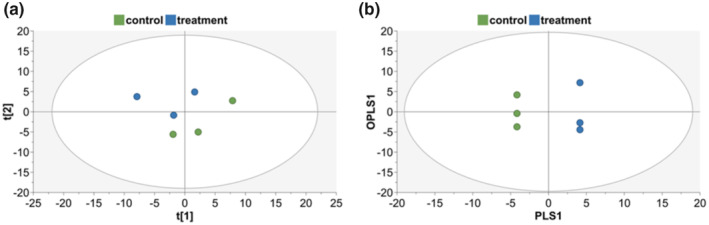
Plots of spectral data using PCA and OPLS‐DA. (a) Clustering by principal components analysis (PCA) is demonstrated for vehicle control (0 ng/mL; green) and TGF‐β1 treatment (10 ng/mL; blue). In (b) orthogonal projections to latent structures – discriminant analysis (OPLS‐DA) plots demonstrate clear separation of control and treated groups using supervised assignment of components.

The data from vehicle controls or 10 ng/mL TGF‐β1‐treated cells at 48 h (each *N* = 3) were calculated using PCA (Figure 4a) and OPLS‐DA (Figure 4b). PCA scores demonstrate clustering with TGF‐β1 treatment, with some separation between control (green) and treatment (blue) groups (Figure 4a; R^2^X = 0.59, Q_2_ = −0.162). When all data were modeled into supervised OPLS‐DA, clear separation between control and treatment groups is demonstrated, based on 82 spectroscopic features (R^2^X and Q_2_ = 1).

Utilizing VIP ≥1.2 as a cut‐off for modeling, twenty‐four resonances were identified. These resonances and their changes following treatment are provided in Table 2 ordered from the highest to lowest contribution to the separation of groups.

**TABLE 2:** Resonance assignments contributing to separation of untreated and TGF‐β1‐treated HK‐2 cell groups according to VIP score.

F2 (ppm)	F1 (ppm)	Resonance assignment	Fold change from control	Treatment
3.21	3.20	Choline	0.88 ± 0.05	↓
3.61	3.27	Myo‐inositol	0.66 ± 0.07	↓
2.33	2.11	Glutamate	0.80 ± 0.16	↓
4.19	3.58	Phosphorylcholine	0.88 ± 0.22	↓
4.11	2.30	n‐Acetyl‐glutamine	0.73 ± 0.20	↓
2.15	3.77	Glutamine/Glutamate	0.86 ± 0.13	↓
3.38	1.51	L‐pipecolic acid	1.17 ± 0.14	↑
1.68	2.99	L‐lysine	1.10 ± 0.12	↑
2.13	2.53	Glutathione (Glutamate moiety)	0.87 ± 0.07	↓
3.58	3.58	Glyceraldehyde	0.89 ± 0.04	↓
4.63	4.63	D‐glucose	1.36 ± 0.05	↑
5.20	4.10	Glucuronic acid	1.22 ± 0.32	↑
4.24	1.31	Fucose/threonine	0.88 ± 0.03	↓
3.35	1.98	Undefined	0.89 ± 0.09	↓
1.51	3.38	Undefined	1.17 ± 0.14	↑
1.99	3.36	Undefined	0.81 ± 0.08	↓
4.32	3.67	Glycerophosphocholine (Choline moiety)	0.75 ± 0.07	↓
4.55	2.93	Glutathione (Cysteine moiety)	0.94 ± 0.07	↓
3.19	3.02	Tyrosine	0.79 ± 0.04	↓
0.88	0.88	Lipid CH_3_	1.04 ± 0.05	↑
1.32	2.01	Lipid B	1.05 ± 0.13	↑
3.77	3.75	Acetylcholine/glucose	1.01 ± 0.03	↓
2.10	3.22	Undefined	1.52 ± 1.18	↑
2.34	2.32	Glutamate/lipid	0.93 ± 0.05	↓

*Note*: Chemicals were ordered by the VIP scores from highest to lowest. The two frequency domains, F1 and F2, are in parts per million (ppm). Resonances were termed “undefined” due to limited information in the HMBD and current literature. Mean ± SD Fold change of treatment from control is displayed. The direction of expression in the treated samples (TGF‐β1 10 ng/mL, 48 h) compared with the vehicle controls (TGF‐β1 0 ng/mL, 48 h) is noted with ↑ = increased and ↓ = decreased.

## 4 DISCUSSION

PTEC contribute to a significant proportion of total cellular mass of the kidney and to kidney function. They contain many large mitochondria for the energy demands of their role of glomerular filtrate reabsorption. With persistent injury, they become key mediators in the development of tubulointerstitial fibrosis and subsequently CKD (Gewin et al., 2017; Grgic et al., 2012; Hewitson et al., 2017; Owens et al., 2022). During the pathogenesis of CKD, PTEC can undergo phenotypical changes that contribute to exacerbate inflammatory and fibrotic states in the kidney, including augmentation of the resident and infiltrating myofibroblast populations (Qi & Yang, 2018). Both the PTEC and myofibroblasts secrete ECM proteins into the interstitial space (Meng et al., 2016). TGF‐β1, a key regulator and primary driver of kidney fibrosis, facilitates these activities by promoting transcriptional and translational activation of pro‐fibrotic and pro‐inflammatory genes (Kim et al., 2018; Meng et al., 2016). The metabolic alterations caused by TGF‐β1 in kidney fibrosis, however, are not well defined. This investigation aimed to increase knowledge of altered metabolites or chemicals in PTEC when exposed to pro‐fibrotic factors.

In our investigation, TGF‐β1 treatment of HK‐2 PTEC induced increased transcript levels of FN and COL4A1. These genes are part of a fibrogenic cascade for kidney fibrosis and are essential components of the ECM. Transcripts for α‐SMA (ACTA2) as a marker of fibrosis were also increased. Under the influence of TGF‐β1, the cellular morphological characteristics of a cobblestone appearance of healthy kidney tubular epithelial cells in culture changed to a spindle‐shaped mesenchymal phenotype that was suggestive of EMT. The results validated this model of kidney fibrogenesis and allowed selection of an optimal treatment concentration of TGF‐β1 of 10 ng/mL and treatment time of 48 h to demonstrate fibrogenesis—these conditions are in line with other publications using TGF‐β1‐treated HK‐2 cells to study fibrogenesis (Grgic et al., 2012; Yu & Bonventre, 2020). Similar characteristics were also identified in biopsy samples from diabetic nephropathy patients with fibrotic kidneys (Chen et al., 2014; Thakur et al., 2015).

The complex cellular processes involved in kidney fibrosis are energy dependent. The increased requirement for ATP is facilitated by the extensive mitochondrial content of PTEC. There is, however, little information on the specific energy pathways that are altered in kidney fibrogenesis, especially involving the proximal tubular epithelium. In hypermetabolic cells, such as PTEC, FAO is the preferred energy source over aerobic and anaerobic respiration due to its greater ATP‐generating ability (Wiener et al., 1987). FAO is a mitochondrial metabolic process whereby fatty acids are catabolized to form the energy intermediate of acetyl‐CoA, which later enters the tricarboxylic acid cycle (TCA) cycle to produce ATP. Whilst validating the fibrosis model, increased transcript levels of FAO‐associated enzyme ACOX1 was demonstrated, supporting the speculation that energy for fibrogenesis is supplied by FAO. Gene expression of the FAO enzyme ACAA1 was also increased, but this analysis was underpowered and should be considered observational rather than definitive. In other studies, FAO‐related mitochondrial enzymes, peroxisome proliferator‐activated receptors, and carnitine palmitoyltransferase I were decreased, and glycolysis was increased in kidney fibrosis (Jang et al., 2020; Kang et al., 2015). The discrepancy may be explained by the differences in fibrosis modeling. Whole kidney tissue would involve the complete microenvironment of tissue fibrosis, whereas a cell culture model, of necessity, focuses on the contribution of selected cell types and pro‐fibrotic initiators, thereby limiting comparisons that can be drawn.

The metabolic changes to fatty acid, glucose, and amino acids in PTEC have also received attention in the context of kidney fibrosis and CKD development, with results demonstrating that metabolic dysregulation and tubulointerstitial fibrosis of CKD are linked. Using a preclinical CKD model of AMP‐activated protein kinase (AMPK) knockout mice subjected to AKI, the modulated AMPK suppressed kidney fibrosis (Wang et al., 2020). AMPK is a central regulator of energy homeostasis and coordinator of cellular metabolic pathways like FAO and glycolysis. However, other recent studies conflict these findings, whereby two AMPK activators, metformin and 5‐aminoimidazole‐4‐carboxamide‐1 riboside, attenuated EMT marker expression, lipid accumulation, and fibrosis in an in vitro albumin‐induced PTEC fibrogenesis model and an in vivo folic acid nephropathy model of fibrosis (Clark & Parikh, 2021; Han et al., 2021; Herzig & Shaw, 2018; Lin & Hardie, 2018). The findings indicate that metabolic dysfunction occurs during kidney fibrosis and that investigation of AMPK in future studies is recommended.

NMR spectroscopy has the potential to monitor progression or success of therapies in CKD investigations (Humphries et al., 2023). The next step in the current investigation was to use ^1^H‐NMR 2D‐COSY in the validated kidney fibrogenesis model with methods established by us in preclinical models of renal carcinoma (Del Vecchio et al., 2022; Urquhart et al., 2023). Using multivariate statistics and a VIP score of ≥1.2, which is a conservative estimate of important metabolites or chemicals contributing to separation of controls and TGF‐β1‐treated HK‐2 cells (Del Vecchio et al., 2022; Urquhart et al., 2023), 24 altered metabolites were recognized.

Decreased levels of myo‐inositol with treatment were accompanied by increased levels of glucuronic acid. The myo‐inositol results are similar to the findings in previous studies using preclinical animal models of CKD (Chang et al., 2015; Cohen et al., 1995; Hsu et al., 2021) and serum samples of CKD patients with multiple aetiologies (Kakkanattu et al., 2022), whereby decreased myo‐inositol was associated with CKD pathogenesis. Myo‐inositol is a molecule involved in glucose and lipid metabolism, and cellular signaling. It is oxidized to produce glucuronic acid, which can act as a substrate for the glucuronate‐xylulose pathway, with subsequent metabolites used in other energy pathway such as the Pentose Phosphate Pathway (PPP) (Smith et al., 2014; Wieman et al., 2007). Upregulation of the PPP was also seen in kidney tissue following ischemia–reperfusion injury, a well‐established risk factor for CKD (Nakagawa & Kang, 2021). However, further exploration of metabolic alterations to the PPP in CKD models is necessary.

Glucose and other substrates involved in glycolysis are known to change in CKD (Chen et al., 2023; Gu et al., 2022; Scantlebery et al., 2021; Stumvoll et al., 1999). In the current study, increased levels of glucose were seen suggesting that glycolysis may be reduced or limited in PTEC during fibrogenesis. This result contrasts with studies using biofluid samples from established instances of CKD: from CKD patients (Hewitson & Smith, 2021); and from the UUO model of kidney fibrosis in mice (Yang et al., 2023), that showed increased glycolysis and the glycolytic products, pyruvate and lactate. Pyruvate and lactate are both detectable by 2D‐COSY but they did not contribute significantly to the separation between control and treatment based on VIP score less than 1.2. Moreover, the gluconeogenesis pathway which occurs in PTEC contains the same metabolites that were altered in our study and recently reported as impaired in CKD (Verissimo et al., 2022). Therefore, the changes observed could indicate flux in either of these counter‐regulated pathways.

The current findings also report decreased metabolite levels of glutamate, glutamine, and glutathione. This agrees with findings from previous literature which reported diminished signatures of glutamate and glutamine in serum samples from patients with kidney failure mediated by immunoglobin A (Sui et al., 2012), a leading cause of CKD. Glutamine can undergo conversion to glutamate, catalyzed by phosphate‐dependent glutaminase, as an important step to the TCA cycle with entry at 2‐oxoglutarate as a metabolic intermediate. The current findings of reduced glutamate suggest that, in early development of kidney fibrosis, this metabolic pathway may be attenuated. Glutamate can be further metabolized to produce glutathione, which is commonly known for its protective role against reactive oxygen species. The decreased levels of glutathione suggest that PTEC may be prone to oxidative stress during fibrogenesis. This speculation corresponds with previous literature that showed that TGF‐β1 enhanced induction of NADPH oxidase‐4, an enzyme involved in the mitochondrial electron transport chain which produces damaging oxygen species and suppresses expression of antioxidant molecules and enzymes (Jiang et al., 2014). These findings support our results that demonstrate aberrant glutamine metabolism.

The strengths of the current investigation were the focused exploration of fibrotic stressor‐mediated metabolic changes in PTEC and how such changes may contribute to the development of kidney fibrosis. Use of ^1^H‐NMR 2D‐COSY was a significant strength in that study design. Unlike 1D spectroscopy that is used, for example, in basic magnetic resonance imaging and spectroscopy, 2D‐COSY allows for unambiguous assignments to chemicals detected in the samples. This novel technology also employs similar principles to that of clinical in vivo magnetic resonance spectroscopy (Ramadan et al., 2012), which allows translatability of the current findings towards development of a potential disease biomarker panel.

Use of the PTEC model was a strength and limitation of this investigation. While the use of the HK‐2 PTEC provided a robust and informative platform for a pilot metabolomics investigation, primary PTEC sourced from healthy kidney tissue may provide a better representation in vitro of fibrotic processes than the established HK‐2 cell line. Another consideration is that the simplicity of a single kidney cell type in our PTEC model limited comparisons with the results of studies of fibrosis using complex whole‐tissue models or biofluids from humans. For future studies using in vitro models of kidney fibrosis, consideration should be made to using multiple cell types of the fibrotic environment with diverse inducers of fibrosis, including pro‐inflammatory molecules, to mimic the in vivo fibrogenic process. It will be important to determine if the findings can be replicated in other in vitro models of CKD and, potentially, be transitioned into in vivo preclinical animal models to further verify our findings. Finally, it remains a priority to explore additional markers of enzymes and substrates involved in the metabolic pathways of FAO, glycolysis, glutamine metabolism, and the PPP, to acquire further evidence of the affected pathways. Introducing specific inhibitors to the in vitro model and investigating their effects on cellular metabolic functions and survival may provide greater insights into the extent of metabolic contributions of such pathways.

In conclusion, this investigation has successfully used a variety of molecular assays to assess and validate an in vitro TGF‐β1‐treated HK‐2 cell model of kidney fibrogenesis using the evidence of EMT, accumulation of ECM proteins, and an altered metabolite profile associated with TGF‐β1‐treated HK‐2 cells using assays that included 2D‐COSY NMR. With refinement and expansion of the current study, we hope to further elucidate the metabolic alterations of PTEC in tubulointerstitial fibrosis, with an aim to contribute to a disease biomarker panel which may eventually be used in vivo for the diagnosis and management of CKD clinically.

## FUNDING INFORMATION

TLRH was supported by the Australian Government Research Training Program Stipend.

## CONFLICT OF INTEREST STATEMENT

The authors have no conflicts of interest to declare.

## ETHICS STATEMENT

No ethical approvals were needed for this study.

## Data Availability

Data will be made available upon request to the corresponding author.
